# Facile Synthesis and Characterization of Palm CNF-ZnO Nanocomposites with Antibacterial and Reinforcing Properties

**DOI:** 10.3390/ijms22115781

**Published:** 2021-05-28

**Authors:** Janarthanan Supramaniam, Darren Yi Sern Low, See Kiat Wong, Loh Teng Hern Tan, Bey Fen Leo, Bey Hing Goh, Dazylah Darji, Fatimah Rubaizah Mohd Rasdi, Kok Gan Chan, Learn Han Lee, Siah Ying Tang

**Affiliations:** 1 Chemical Engineering Discipline, School of Engineering, Monash University Malaysia, Bandar Sunway 47500, Selangor Darul Ehsan, Malaysia; janarthanan.supramaniam@monash.edu (J.S.); darrenl333.dl@gmail.com (D.Y.S.L.); see.wong@monash.edu (S.K.W.); 2 Clinical School Johor Bahru, Jeffrey Cheah School of Medicine and Health Sciences, Monash University Malaysia, Johor Bahru 80100, Johor Darul Ta’zim, Malaysia; loh.teng.hern@monash.edu; 3 Novel Bacteria and Drug Discovery Research Group (NBDD), Microbiome and Bioresource Research Strength (MBRS), Jeffrey Cheah School of Medicine and Health Sciences, Monash University Malaysia, Bandar Sunway 47500, Selangor Darul Ehsan, Malaysia; 4 Faculty of Medicine, University of Malaya, Kuala Lumpur 50603, Malaysia; beyfenleo@um.edu.my; 5 Nanotechnology and Catalysis Research Centre, University of Malaya, Kuala Lumpur 50603, Malaysia; 6 Biofunctional Molecule Exploratory Research Group (BMEX), School of Pharmacy, Monash University Malaysia, Bandar Sunway 47500, Selangor Darul Ehsan, Malaysia; goh.bey.hing@monash.edu; 7 College of Pharmaceutical Sciences, Zhejiang University, Hangzhou 310058, China; 8 Health and Well-Being Cluster, Global Asia in the 21st Century (GA21) Platform, Monash University Malaysia, Bandar Sunway 47500, Selangor Darul Ehsan, Malaysia; 9 Malaysian Rubber Board Engineering and Technology Division, RRIM, Sungai Buloh 47000, Selangor Darul Ehsan, Malaysia; dazylah@lgm.gov.my (D.D.); rubaizah@lgm.gov.my (F.R.M.R.); 10 Institute of Biological Sciences, Faculty of Science, University of Malaya, Kuala Lumpur 50603, Malaysia; 11 International Genome Centre, Jiangsu University, Zhenjiang 212013, China; 12 Advanced Engineering Platform, School of Engineering, Monash University Malaysia, Bandar Sunway 47500, Selangor Darul Ehsan, Malaysia; 13 Tropical Medicine and Biology Platform, School of Science, Monash University Malaysia, Bandar Sunway 47500, Selangor Darul Ehsan, Malaysia

**Keywords:** cellulose nanofiber, palm biomass, zinc oxide, ultrasonic, antibacterial

## Abstract

Cellulose nanofibers (CNF) isolated from plant biomass have attracted considerable interests in polymer engineering. The limitations associated with CNF-based nanocomposites are often linked to the time-consuming preparation methods and lack of desired surface functionalities. Herein, we demonstrate the feasibility of preparing a multifunctional CNF-zinc oxide (CNF-ZnO) nanocomposite with dual antibacterial and reinforcing properties via a facile and efficient ultrasound route. We characterized and examined the antibacterial and mechanical reinforcement performances of our ultrasonically induced nanocomposite. Based on our electron microscopy analyses, the ZnO deposited onto the nanofibrous network had a flake-like morphology with particle sizes ranging between 21 to 34 nm. pH levels between 8–10 led to the formation of ultrafine ZnO particles with a uniform size distribution. The resultant CNF-ZnO composite showed improved thermal stability compared to pure CNF. The composite showed potent inhibitory activities against Gram-positive (methicillin-resistant *Staphylococcus aureus* (MRSA)) and Gram-negative *Salmonella typhi* (*S. typhi*) bacteria. A CNF-ZnO-reinforced natural rubber (NR/CNF-ZnO) composite film, which was produced via latex mixing and casting methods, exhibited up to 42% improvement in tensile strength compared with the neat NR. The findings of this study suggest that ultrasonically-synthesized palm CNF-ZnO nanocomposites could find potential applications in the biomedical field and in the development of high strength rubber composites.

## 1. Introduction

Cellulose has a firm grip as an inexhaustible organic material that could feed the growing demand for green reinforcement nanofillers in various nanocomposite materials. Cellulose is comprised of linear chains of β-D glucopyranosyl units linked by β-(1,4) glycosidic bonds [1]. Cellulose nanofiber (CNF) is a popular biopolymer that is used extensively in various industries, such as in textiles, papers, and pharmaceuticals, due to their exceptional biocompatibility and biodegradability [2,3]. CNFs can be obtained from a wide variety of natural sources, including algae, bacteria, and plants [4,5]. 

Oil palm trees are a valuable agricultural crop in Malaysia that provides multiple benefits to the economic growth of the country [6]. The production of palm oil generates a substantial amount of biomass waste, such as oil palm empty fruit bunches (OPEFB) [6]. On average, 15 million tons per hectare of OPEFB biowaste is produced in Malaysia annually [7,8]. The high availability of OPFEB provided us a substantial reason to choose OPEFB isolated CNF for this study. Moreover, creating higher value-added products could potentially prevent OPEFB from ending up in the natural environment as waste.

Weak interfacial adhesion and the ease of decomposition caused by bacterial attacks often impede promising potential applications of CNF [9,10]. CNF-loaded metallic nanoparticles have been widely explored, such as nanofillers in wound dressing material [11] and in rubber reinforcement [5]. The abundant hydroxyl group availability for surface functionalization, large aspect ratio, and percolation network formation of CNF facilitates its modification with metallic nanoparticles [12]. Zinc oxide (ZnO) has attracted wide interest in the field of nanomaterials due to its high stability, photocatalytic activity, antibacterial activity, and non-toxicity [13,14]. 

The agglomeration of ZnO is a limitation during the synthesis of cellulose nanofiber-zinc oxide (CNF-ZnO) nanocomposites due to its high surface area, and this could cause dispersion issues of the metallic particles in the CNF network [13]. The pH of the reaction solution plays a crucial role in controlling the crystal size and dispersion state of ZnO nanoparticles (NPs) [15]. This is because hydroxyl (OH-) ions strongly influence the polymerization of metal-oxygen bonds during the reaction phase that produces the ZnO nanostructures [16]. Other than pH, the synthesis method also plays a vital role in the uniform dispersion of ZnO on CNF matrices. One of those proposed methods involves the utilization of ultrasonic treatment. 

Ultrasonication could enhance the uniform dispersion of ZnO NPs [17], which would be capable of enhancing the tensile strength of the formed nanocomposites as well as the release kinetics of antibacterial elements [13]. However, the synthesis process in producing this CNF-ZnO nanocomposite is time-consuming [10,14,18,19,20], which may impede the application of this strategy for pilot-scale production. Although the importance of pH on the formation of ZnO NPs via ultrasound treatment has been extensively explored, its influence on ZnO NP transfer toward the surface of CNFs using ultrasound-assisted co-precipitation methods and their subsequent antimicrobial activities remains unclear.

In this study, we report, for the first time, the feasibility of preparing a multifunctional CNF-ZnO nanocomposite with dual antibacterial and reinforcing properties via a facile and efficient ultrasound-assisted co-precipitation approach. We characterized and examined the microstructural properties, thermal stability, and bactericidal potential of ZnO-embedded CNF isolated from OPEFB. In addition, the influence of the reaction solution pH on formation and morphology of ZnO on CNF matrix under ultrasonic field was systematically investigated and discussed. 

In the current work, we first prepared the CNF-ZnO nanocomposite by dispersing the CNF and zinc acetate dihydrate in ultrapure water using physical agitation and, subsequently, subjected it to ultrasonic treatment. The addition of precursor into the mixture led to the presence of more hydroxyl groups on the CNF surface, which provides extra capacity for Zn^2+^ ion attachment. Positively charged zinc ions will be attracted to the oppositely charged CNF during the mixing process. 

In addition, the introduction of the precursor material initiates hydrolysis and the condensation processes of zinc salts that will eventually progress to the nucleation of ZnO NPs. The approach reported in this paper offers a swift ultrasonic-assisted hydrothermal synthesis route, with a suitable pH environment for CNF-ZnO nanocomposite fabrication. A comprehensive antibacterial study of the as-synthesized CNF-ZnO was carried out by disc diffusion. Finally, the synthesized CNF-ZnO was incorporated into natural rubber (NR) latex to produce high strength CNF-ZnO-reinforced natural rubber (NR/CNF-ZnO) composite films, and its mechanical improvement was quantified.

## 2. Results and Discussions

### 2.1. Formation Mechanism of ZnO NPs on CNF Matrix

First, zinc acetate dihydrate (Zn(CH_3_COO)_2_·2H_2_O) was dispersed in water to generate Zn^2+^ ions. CNF was then added into the Zn(CH_3_COO)_2_ solution for the pre-nucleation process to produce ZnO NPs. The hydroxyl groups on cellulosic fiber surface became negatively charged due to the ionization process in the solution, which provided active sites for the Zn^2+^ ions [21]. The embedment of Zn^2+^ ions on the cellulosic fibers surface for the pre-nucleation occurred due to electrostatic attraction [10]. The addition of sodium hydroxide (NaOH) provided an alkaline condition with OH^−^ anions, which reacted with the Zn^2+^ cations produced from (Zn(CH_3_COO)_2_·2H_2_O) and generated primary growth units of ZnO ([Zn(OH)_4_]^2-^ ions). 

**Figure 1:**
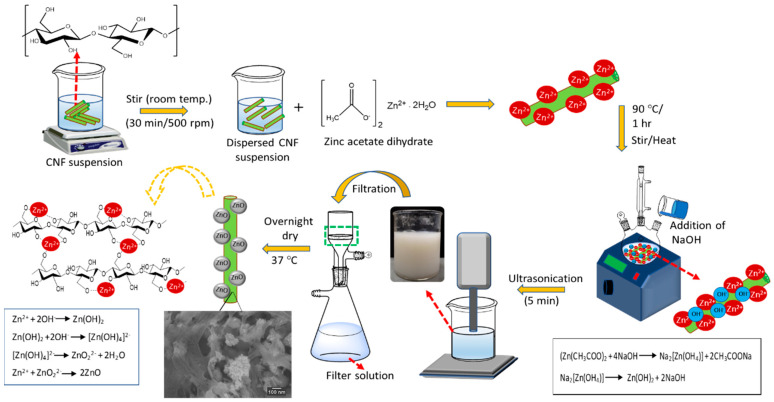
Sonochemical preparation of the cellulose nanofiber-zinc oxide (CNF-ZnO) nanocomposite.

In other words, the alkaline conditions resulted in greater ionization of cellulosic chains for ZnO nucleus formation and the simultaneous formation of hydrated hydroxides via hydrolysis reactions [22,23]. The ZnO NP generation occurs when Zn(OH_4_)^2-^ decomposes at elevated temperatures [10]. Ultrasonication creates a pressured atmosphere, which induces the deep growth of ZnO NP on the surface of cellulosic fibers. The sonochemical mechanism of ZnO NP embedment on a cellulosic fiber surface is illustrated in Figure 1.

### 2.2. Morphology of CNF-ZnO Nanocomposite

**Figure 2:**
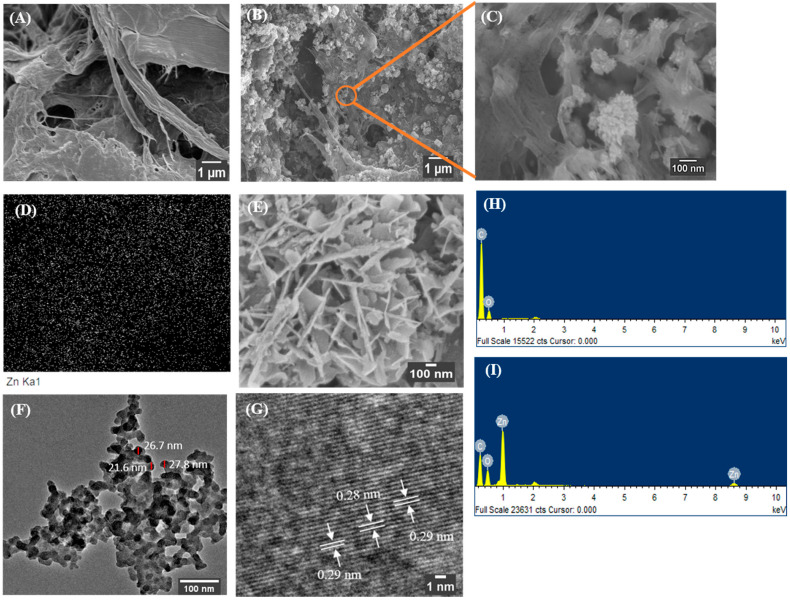
FESEM, EDX, and TEM analysis. (**A**) Pure CNF, (**B**) CNF-ZnO nanocomposite (pH 10), (**C**) Focused image of CNF-ZnO (**D**) EDX mapping of CNF-ZnO (pH 10), (**E**) ZnO NP synthesized at pH 10, (**F**) TEM of ZnO from nanocomposite, (**G**) Lattice spacing of ZnO, (**H**) EDX Spectrum of pure CNF, and (**I**) EDX Spectrum of CNF-ZnO nanocomposite (pH 10).

Electron micrographs of unmodified CNF and as-synthesized CNF-ZnO nanocomposites are presented in Figure 2. The surface structure of pure CNF (Figure 2A) appeared to be smooth showing no presence of ZnO NPs. The field-electron scanning electron microscopy (FESEM) image in Figure 2B,C shows the distribution of ZnO NPs in the CNF matrix, indicating its embedment. The structure of ZnO NPs embedded in the CNF matrix appeared as flake-like particles. The energy dispersive X-ray (EDX) mapping shown in Figure 2D interprets the uniform arrangement of ZnO NPs (represented by white specks) in the CNF matrix. The ZnO NPs morphology synthesized at pH 10 also appear to have the flake-like morphology (Figure 2E), which corresponds to ZnO NPs on the CNF surface (Figure 2C). 

The average particle size of ZnO NPs measured using transmission electron microscopy (TEM) was 25.4 ± 3 nm as indicated in Figure 2F. At high-resolution TEM analysis as in Figure 2G, the synthesized ZnO NPs had a lattice spacing of 2.9 Å, which closely corresponded to the ‘d’ value obtained from the XRD results of 2.8 Å, from the Joint Committee on Powder Diffraction Standards (JCPDS) card no. 36-1451. The lattice spacing observed was indexed to the (100) plane of the ZnO NPs structure. Furthermore, the EDX spectra in Figure 2I proves the formation of ZnO NPs by providing the desired peaks associated with zinc and oxygen elements, while pure CNF showed an absence of elemental zinc in Figure 2H.

As shown in Supplementary Figure S1A, the ZnO NPs agglomerated distinctly on the CNF surface. A study conducted by Bian, et al. [24] highlighted the role of pH dissolution and the aggregation of ZnO NPs. As the pH approaches the reported zero point of charge for ZnO (9.2), the repulsive interaction between the NP decreases [24]. ZnO NPs showed lower solubility and were more stable at pH 9–11, respectively. Increasing the suspension above pH 12 causes an equilibrium between hydroxyl complexes (Zn(OH)_3_^−^ and Zn(OH)_4_^2−^) and ZnO NPs, which leads to further decreases in dissolution [25], contributing to severe agglomeration of the ZnO NPs on the cellulose fiber surface. 

Generally, the ZnO NPs were observed to be well dispersed in the CNF network when the synthesized environment changed to pH 8 (Figure S1C) and pH 10 (Figure S1B). This was due predominantly to electrostatic attractions and the polar nature of ZnO NPs. The ZnO tends to attract and attach to the negatively charged oxygen groups, such as oxygenated side groups and oxygenated terminal groups of the CNF [26,27]. Within the acidic scale, there was less attachment of ZnO NPs on the CNF surface as shown in Figure S1D,E. 

The reason could be due to the acidic environment, which likely induces the dissolution of ZnO from a direct proton attack of the ZnO surface [24]. More soluble ionic forms, such as Zn^2+^ and Zn(OH)^+^, will be formed at pH values below 6. Based on this study, basic conditions of pH 8–10 were most suitable for the embedment of ZnO NPs on the CNF network. Detailed information on the microstructure of pure CNF and CNF-ZnO nanocomposite was further investigated using FESEM and TEM analyses.

### 2.3. Chemical Composition of CNF-ZnO Nanocomposite

**Figure 3:**
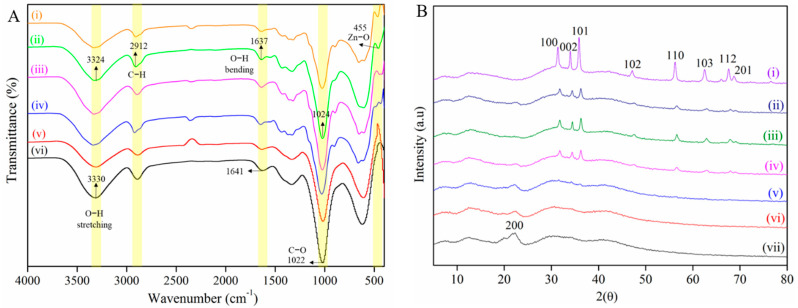
FTIR spectra (**A**) for (i) CNF-ZnO pH 12, (ii) CNF-ZnO pH 10, (iii) CNF-ZnO pH 8, (iv) CNF-ZnO pH 6, (v) CNF-ZnO pH 4, and (vi) pure CNF; (**B**) XRD diffractograms for (i) ZnO NP, (ii) CNF-ZnO pH 12, (iii) CNF-ZnO pH 10, (iv) CNF-ZnO pH 8, (v) CNF-ZnO pH 6, (vi) CNF-ZnO pH 4, and (vii) pure CNF.

FTIR spectroscopy was used to study the chemical interactions between ZnO NPs and the CNF matrix with the spectra as shown in Figure 3A. The peak of O-H stretching vibration (3331 cm^−1^) of pure CNF shifts to lower wavenumber (3324 cm^−1^) from CNF-ZnO, indicating the interaction between the cellulosic matrix and ZnO NPs [28]. The peaks of hydroxyl groups (3331 cm^−1^) and C-H vibrations (2912 cm^−1^) of cellulose show intensity reduction in the CNF-ZnO spectrum due to physical interactions between the cellulose matrix and ZnO NPs [29]. 

We observed that the wavenumber shifts between pure CNF at 1641 cm^−1^ and CNF-ZnO at 1637 cm^−1^ were caused by hydrogen bond splitting due to the embedment of ZnO NPs in the cellulose matrix [19,30]. The stretching of C-O bonds was displayed at 1022 cm^−1^ and 1024 cm^−1^ for pure CNF and the nanocomposite, respectively [19]. A new peak was observed for the nanocomposite at 455 cm^−1^, which was ascribed to the stretching vibration of the Zn-O bonds, thus, providing evidence of the successful embedment of ZnO NPs on the CNF matrix to form CNF-ZnO nanocomposites [14]. In general, vibrational peaks for Zn-O bond stretching were indicated in the region between 400 cm^−1^ and 600 cm^−1^ [31,32].

The XRD pattern of CNF-ZnO shown in Figure 3B was further evidence of ZnO NP embedment on the OPEFB-isolated CNF. This provides the characteristics of ZnO NP XRD diffraction patterns with diffraction peaks at 2θ with the values, 37.1°, 34.4°, 36.2°, 47.5°, 56.6°, 62.8°, 67.7°, and 69.1° corresponding to (100), (002), (101), (102), (110), (103), (112), and (201), respectively. The results obtained for the ZnO NPs match the JCPDS card no. 36-1451. The XRD peaks obtained show the formation of the wurtzite structure for the ZnO NPs [33]. Additional peaks in the XRD spectra for CNF-ZnO were present in comparison with the pure CNF, indicating the successful binding of ZnO NPs on CNF without any impurities. The results obtained match with other types of ZnO NP-loaded nanocomposites, such as ZnO-cellulose cotton linter pulp [34] and ZnO-cellulose cotton fabric [21].

### 2.4. Thermal Behavior of the CNF-ZnO Nanocomposite

**Figure 4:**
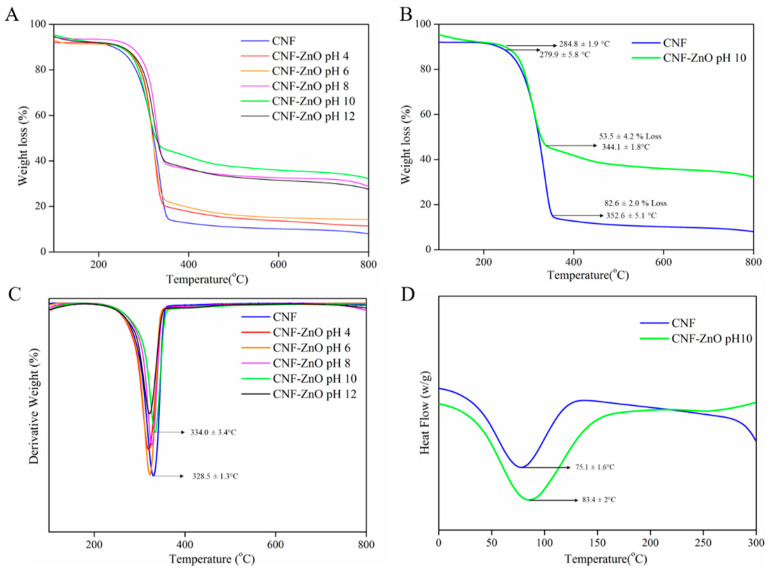
(**A**) TGA analysis for the all samples, (**B**) Detailed TGA analysis for pure CNF and CNF-ZnO pH 10, (**C**) DTG curve for pure CNF and CNF-ZnO pH 10, and (**D**) DSC analysis for pure CNF and CNF-ZnO pH 10.

The TGA curves of the CNF-ZnO nanocomposite prepared at different pH values are presented in Figure 4A. We observed that the onset temperature of pure CNF and CNF-ZnO prepared at pH 10 occurred at 280 and 285 °C, respectively in Figure 4B. The degradation peak of pure CNF was increased from 329 to 334 °C upon the incorporation of ZnO NPs, as depicted in Figure 4C. The improved heat resistance of the incorporated ZnO NPs could be ascribed to the enhancement of thermal stability in the fabricated CNF-ZnO nanocomposite [14].

Figure 4D shows the DSC curves for both pure CNF and CNF-ZnO samples. The incorporation of ZnO NPs into the cellulose network increased the melting temperature (T_m_) from 75.1 to 83.4 °C. The observed increases in T_m_ were attributed to the presence of metallic ZnO in the composite materials. The obtained result was in agreement with the reported work by Abdalkarim, et al. [14], where the prepared biopolyester shifted to higher temperatures with the addition of a ZnO-cellulose nanocomposite. The TGA and DSC findings indicated that the CNF-ZnO nanocomposite had better thermal stability compared with the pure CNF.

### 2.5. Release Properties of Zn^2+^ from the CNF-ZnO Nanocomposite

**Figure 5:**
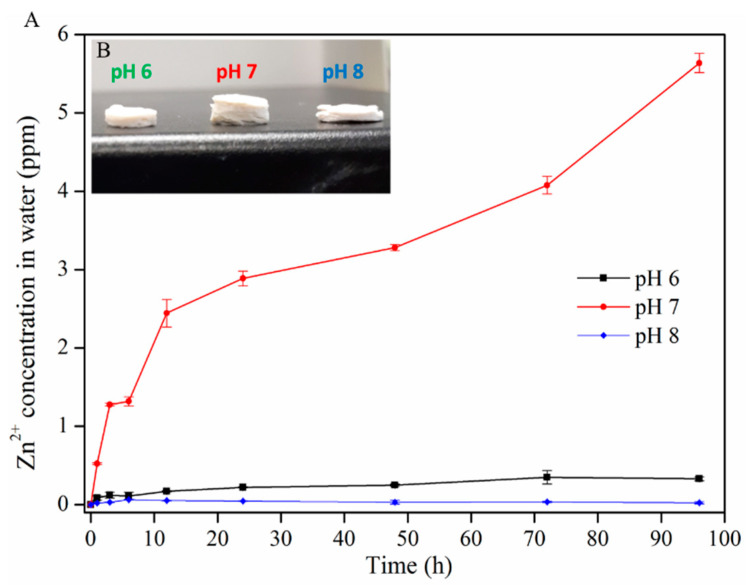
(**A**) Release profile of Zn^2+^ ions from the CNF-ZnO composite at different pH values. ((**B**) inset) CNF-ZnO specimens subjected to swelling at different conditions of pH 6, pH 7, and pH 8.

Figure 5A shows the effect of the pH on the release behavior of Zn^2+^ ions from the CNF-ZnO nanocomposite. At pH 7, Zn^2+^ ions experienced a higher release rate followed by sustained release at pH 6 and 8, eventually reaching 1.69 ppm after 96 h. Similar minimal releases of Zn^2+^ ions were observed at pH values of 6 and 8. The increased swelling of the CNF-ZnO nanocomposite at pH 7 (Figure 5B) allowed more space for the release of entrapped Zn^2+^ ions from the CNF matrix. The osmotic pressure in the CNF matrix created by the surface electric charge of ZnO was balanced by the absorption of greater amounts of water. 

A similar explanation was provided by George, et al. [35] for chitosan–cellulose hydrogel swelling phenomena. Increasing or lowering the pH to 8 and 6 did not lead to significant swelling of the composite, as evidenced in Figure 5B. The results obtained in this study are in agreement with the reported work by Yadollahi et al., who showed that the fabricated cellulose/ZnO nanocomposite swelling capacity increased as the pH level changed from 2–7, but decreased when exceeding pH 7 [36]. An excess Na^+^ cation concentration from NaOH produces the shielding effect and blocks complete intermolecular repulsion. This further restrains the tangled molecular chain of the nanocomposite [36,37]. Thus, the swelling of the CNF is reduced and prevents the release of the entrapped ZnO in the closely packed CNF matrix [38]. 

The total Zn^2+^ ions present on the CNF was 644.7 ppm for the nanocomposite. The total Zn^2+^ ion concentration embedded on the CNF was estimated based on simple stoichiometric calculations as shown in the Supplementary information. The highest cumulative Zn^2+^ ion release was found at pH 7 (1.69 ppm after 96 h), which was very low compared to the total estimated concentration of Zn^2+^ ions embedded on the CNF. The minimal release of Zn^2+^ ion affirms the strong electrostatic interactions of ZnO NPs with CNF. The uncontrollable release of Zn^2+^ ions may pose serious risk to human health and the environment due to its potentially toxic effects [39]. However, the reduced duration of the CNF-ZnO nanocomposite synthesis in our work did not induce adverse effects regarding Zn^2+^ ion release from the nanocomposite.

### 2.6. Antibacterial Activity of CNF-ZnO Nanocomposite

**Figure 6:**
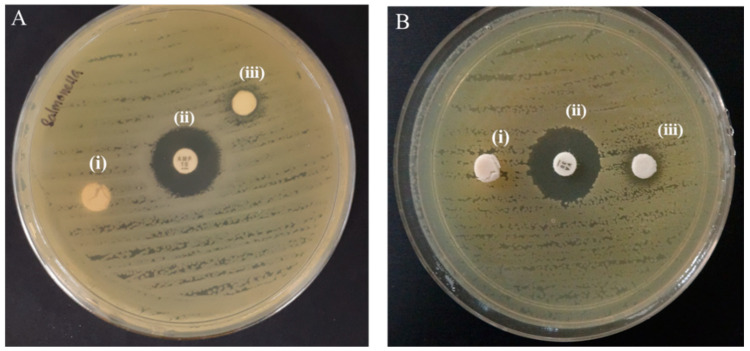
(**A**) Disc diffusion test with *S. typhi* and respective discs (i) pure CNF, (ii) vancomycin, and (iii) CNF-ZnO pH 10. (**B**) Disc diffusion test with MRSA and respective discs (i) pure CNF, (ii) vancomycin, and (iii) CNF-ZnO pH10.

Figure 6A,B presents the antibacterial susceptibility test of the as-synthesized CNF-ZnO nanocomposite by the disc diffusion method. The results demonstrated that the ultrasonically-prepared CNF-ZnO composite was able to inhibit bacterial growth with inhibition zones recorded at 7.68 ± 0.08 mm and 9.77 ± 0.41 mm in diameter for *Salmonella typhi* (*S. typhi*) and methicillin-resistant *Staphylococcus aureus* (MRSA), respectively. As expected, there was no inhibition zone observed for the pure CNF sample as the control. 

This result also correlates well with the release properties of CNF-ZnO (Figure 5), whereby the slow release of zinc ions and the thick attachment of the nanoparticles (Figure 2C) was sufficient to interact with the bacteria and cause growth inhibition. According to the Standard Antibacterial test “SNV 195920-1992”, antibacterial agents can be defined as materials that can demonstrate inhibition areas to bacterial growth greater than 1 mm [18,40]. The disc diffusion test showed that the CNF-ZnO nanocomposite exhibited better inhibitory action against MRSA than against *S. typhi*. Similar observations were also reported in earlier literature regarding cellulose-based ZnO nanocomposites [28,41].

The difference in inhibitory effects could be attributed to the different structural variations in bacterial cell walls, as it is well documented that the Gram-negative bacterial strains possess complex chemicals and structural arrangements with outer membranes comprising lipopolysaccharides, which impart protective properties against toxic and harmful environments [42,43,44]. On the other hand, Gram-positive bacterial strains consist of layers of peptidoglycan with lipoteichoic and teichoic acids, which, in turn, makes them more vulnerable to certain antibacterial agents [42].

The reduction in viable cell count could be explained by the fact that the ZnO NPs possess the ability to generate reactive oxygen species (ROS), such as superoxide radicals, singlet oxygen species, and hydroxyl radicals, which are responsible for the antibacterial activity of the as-synthesized nanocomposite [45,46]. The ROS produced by the ZnO NPs subsequently resulted in DNA damage in the bacterial species through the induction of oxidative stress and bacterial protein damage associated with transmembrane electron transport impairment [47]. 

MRSA has been reported to develop resistance toward vancomycin, which is the current drug of last resort, thus, posing a great challenge to the current healthcare system [48]. Therefore, these CNF-ZnO nanocomposites with anti-MRSA properties could be a promising alternative material for biomedical applications.

### 2.7. Tensile Strength and Heat Resistance Characteristics of NR/CNF-ZnO Composite Film

**Figure 7:**
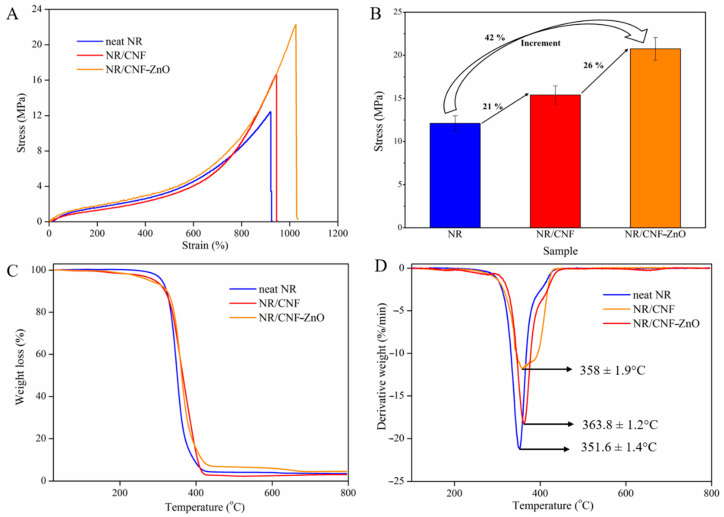
(**A**) Stress–strain curves; (**B**) Tensile strength increment; (**C**) TGA analysis; and (**D**) DTG curves of the neat NR, NR/CNF, and NR/CNF-ZnO composites.

The use of conventional nanofillers, such as carbon black and silica, to enhance the tensile strength has its downsides from an environmental perspective [5]. Hence, cellulosic materials have attracted interest as fillers to replace these conventional fillers [49]. As shown in Figure 7A, the loading of pure CNF in NR resulted in a slight increase in the tensile strength from 12.4 (neat NR) to 16.6 MPa. The presence of hydroxyl groups with high polarity and hydrophilicity of the CNF surface makes the dispersion of cellulosic fibers in the NR network difficult [10,49]. 

The fabricated NR/CNF-ZnO composite film exhibited an improved tensile strength of 22.3 MPa compared to the neat NR and NR/CNF composite. An increase of 42% compared to the neat NR and a 26% strength improvement over NR/CNF was obtained upon incorporation of CNF-ZnO in the NR network as shown in Figure 7B. These findings show that CNF-ZnO has great potential for improving the tensile strength of NR, which could be contributed to by the presence of additional ZnO NPs in NR composites, in addition to those added as parts of the chemical formulation (Table 1). Similar results were obtained by Li et al. [10], whereby an increase of 37.6% tensile strength was recorded for NR reinforced with ultrasonicated cellulosic fibers with longer hours of synthesis. In our findings, the CNF was subjected to a total of 10 min of ultrasonic treatment and an hour of heating to fabricate the CNF-ZnO nanocomposite.

Based on Figure 7C,D, the reduced synthesis duration used in this study did not adversely affect the thermal properties of the NR/CNF-ZnO composite film. The thermal degradation of NR/CNF and NR/CNF-ZnO showed a slight decrease in the degradation onset temperature (Figure 7C). Similar curves have been documented regarding an NR network with CNF, where there was a reduction in the weight loss before the main NR degradation could be observed [50]. The decline of the degradation onset temperature could be ascribed to the lower onset degradation of cellulosic fibers compared to NR [51]. 

However, as the temperature increased, the NR containing CNF-ZnO showed better overall thermal stability compared with the neat NR and NR/CNF, as observed by the shift in the degradation peak to a higher temperature (Figure 7D). The peak shifted from 352 °C for neat NR to 364 °C for NR/CNF-ZnO. The improved degradation temperature can be attributed by the partial shielding of the rubber network by cellulosic fibers and the increased amount of cross-links facilitated by the presence of ZnO [50]. The ability of ZnO to serve as a common activator in the rubber curing process and a reinforcing agent in the NR polymer network [52] as well as having excellent heat resistance properties contributed to the enhanced tensile strength and thermal stability of the NR/CNF-ZnO composite film.

## 3. Materials and Methods

### 3.1. Materials

The CNF used in this study were supplied by the Nanotechnology and Catalysis Research Centre, University of Malaya (Kuala Lumpur, Malaysia). The NR latex was contributed by the Rubber Research Institute Malaysia (RRIM) (Kuala Lumpur, Malaysia). The nanofiber average diameter (~25 nm) was pre-determined. Ethanol (AR grade) was purchased from R&M Chemicals (Syarikat Saintifik Jaya, Selangor, Malaysia). The precursor, sodium hydroxide (NaOH), and zinc ion source, zinc acetate dihydrate (Zn(CH_3_COO)_2_·2H_2_O), were procured from Sigma-Aldrich, MO, USA. A Mili-Q^®^ Plus (Millipore, Billerica, MA, USA) apparatus was utilized to produce ultrapure water (18.2 MΩ∙cm). The ultrasonic horn system (NexTgen, Sinaptec, Lezennes, France) with a frequency of 20 kHz and power of 100 W was used with a fixed setting during the synthesis process. It was set with an alternating pulse mode with a 15-s On duration followed by a 10-s Off duration.

### 3.2. Sonochemical Preparation of CNF-ZnO Nanocomposite

Approximately 1.00 g of CNF was measured and mixed in 200 mL of ultrapure water. We then added 0.44 g of Zn(CH_3_COO)_2_·2H_2_O to the CNF mixture under magnetic stirring at a speed of 500 rpm for 1 h, followed by ultrasonic treatment for 5 min. Next, the mixture was transferred into a three-necked refluxing apparatus equipped with a Liebig condenser and was refluxed at 90 °C for 1 h. The thermal energy in the co-precipitation method is vital to force the hydrolysis process and lead to the formation of ZnO [53]. A digital thermometer (Digi Sense, 20250-01, IL, USA) was used to monitor the temperature of the mixture. Then, 100 mM of NaOH was added to the mixture, and the pH of the mixture was adjusted to pH 10 using 1 M NaOH and 1 M HCl solutions. 

The mixture was stirred at a speed of 500 rpm continuously for 30 min and, subsequently, subjected to ultrasonic treatment for 5 min. The ultrasonicated mixture was then filtered using a standard vacuum filtration setup. After the filtration process, the residue (filter cake) was rinsed with ethanol and distilled water for several cycles to remove excess chemicals. The final product (nanocomposite) of the filtration process was dried overnight, while the filtrate was collected for further ICP-OES analysis. The dried CNF-ZnO nanocomposite was cut into disc-shaped samples with a diameter of approximately 5 mm using a puncher for further characterization. 

### 3.3. Fabrication of NR/CNF-ZnO Composite Film

An overhead mechanical stirrer was used to stir the NR latex for 30 min at 70–80 rpm prior to the addition of the chemical formulation as shown in Table 1. The NR latex was continuously stirred during the addition of chemical agents and CNF-ZnO nanocomposite. The mixture was then homogenized using a dispersing tool (S 25N—18 G Dispersing Element, IKA, Staufan, Germany) for approximately 5 min at 6000 rpm. The foam formed during the dispersion was skimmed using a clean Whatman filter paper. The NR latex was continuously stirred at low speed and kept enclosed at room temperature, overnight. 

The NR latex was subsequently poured on glass petri dishes (90 mm diameter) to fabricate the NR/CNF-ZnO composite films. The NR-filled glass petri dishes were heated at 50–60 °C in an oven until the NR latex was dried. The dried samples were then leached in hot water at 70 °C to remove any excess chemicals that might be present on the sample surface. After the leaching process, the sample was further cured in the oven for another 20 min at 110–115 °C. The NR/CNF-ZnO composite film was then stripped slowly from the glass petri dish. The steps were repeated with the addition of pure CNF to fabricate NR/CNF composite films and without any foreign fillers to produce neat NR films.

### 3.4. Characterization of Synthesized CNF-ZnO Nanocomposite

Morphological analysis was performed using a field emission scanning electron microscope (FESEM, Oxford-Horiba Inca XMax50, Hitachi SU8010, Tokyo, Japan) at 5 kV. Elemental analysis was carried out to further support the presence of ZnO on the CNF matrix using energy-dispersive X-ray spectroscopy (EDX) at an accelerating voltage of 15 kV. High-resolution transmission electron microscopy (HR-TEM, FE Tecnai G2 20 S-Twin, FEI Company, OR, USA) was used to measure the particle size of ZnO NPs.

The chemical composition of the CNF-ZnO nanocomposite was investigated using Fourier-Transform Infrared (FTIR) spectroscopy, Varian 600-IR series (Varian, Mulgrave, Australia) over the range of 400–4000 cm^−1^, and the spectrum was recorded over 32 scans.

X-ray diffraction (XRD) analysis of the nanocomposites was performed using a diffractometer (Bruker D8 Discover, MA, USA) equipped with LynxEye and Vantec-500 detectors and operating at 40 kV and 40 mA with Cu Kα radiation (1.5418 Å). The study was conducted at a 2θ angle ranging from 5° to 80° at a scan rate of 2° per minute and a step size of 0.02°.

A thermogravimetric analyzer (TGA, Q50, TA Instruments, DE, USA) was used to study the thermal stability behavior and weight loss profiles of the samples. The samples were analyzed at temperatures ranging from 30 to 900 °C with a heating rate of 5 °C per minute in a constant airflow rate of 50 mL per minute. A TA Instrument, DSC 25, DE, USA was utilized for the DSC analysis in this study. The dried sample was weighed (5 mg) and sealed in an aluminum sample pan. It was subsequently heated up to 300 °C at a heating rate of 5 °C per minute with 5 min holding time at 250 °C, followed by a temperature decrease to 25 °C at a cooling rate of 10 °C per minute.

The zinc ion content in the CNF before and after treatment was measured using inductively coupled plasma-optical emission spectrometry (ICP-OES) (Perkin Elmer Optima 8000, MA, USA) with a 0.001 ppm metal ion detection limit. The measurement technique is illustrated in Figure S2. The initial collected solution (filtrate) from the filter process of CNF-ZnO nanocomposite formation was used in this analysis. We diluted 1 mL of the filtrate in 1000 mL of water and agitated this solution via magnetic stirring at 500 rpm prior to the analysis. Approximately 10 mL of the diluted effluent was transferred to a 50 mm centrifuge tube and placed on a sample tray for the ICP-OES analysis. The zinc ion content (Z) in the CNF was determined from the following Equation (1):

(1) $${Z=C}_{\mathrm{initial}}{\;-\;(C}_{\mathrm{final}}\;\times \;1000)$$

The Zn^2+^ ion release profile from the prepared CNF-ZnO nanocomposite was measured using the same ICP-OES system with a 0.001 ppm metal ion detection limit. The disc-shaped samples were immersed in three types of solutions, namely acidic (pH 6), neutral (pH 7), and basic (pH 8). The immersing solution was prepared using phosphate buffer solution, and the pH was adjusted using 0.01 M of NaOH and 0.01 M of HCl. 

The samples were immersed in 20 mL of buffer solution in individual glass vials at room temperature. Approximately 1 mL of solution from each vial was collected at pre-determined intervals (0, 1, 3, 6, 12, 24, 48, 72, and 96 h). The collected aliquots were diluted in 10 mL of ultrapure water and analyzed using ICP-OES to determine the amount of Zn^2+^ ions released under different pH conditions. The release kinetics study was performed in triplicates per set. To balance the constant medium volume, an equivalent volume of fresh solution was added into the glass vial after each sampling cycle.

### 3.5. Antibacterial Assay

The antibacterial activity of the samples was tested against Gram-positive bacteria, methicillin-resistant *Staphylococcus aureus* (MRSA) (ATCC 44300), and Gram-negative bacteria, *Salmonella typhi* (*S. typhi*) (ATCC 19430) using a disc diffusion assay. Briefly, the test sample was cut into disc-shapes with a 6-mm diameter and placed on Mueller–Hinton agar (HiMedia, Mumbai, India), which was inoculated with an adjusted overnight culture of bacteria cells (~1 × 10^8^ colony forming units (CFU)/mL). The plate was incubated for 24 h at 37 °C. Vancomycin (30 μg) discs (Oxoid, Hampshire, UK) were applied as positive controls, while pure CNF discs were used as negative controls. The assay was performed in triplicates per set. 

### 3.6. Tensile Tests of NR Composite Films

The stress–strain curves of the samples were determined using a universal testing machine (INSTRON 5966, England, UK) at crosshead speed of 500 mm per minute. The standard specimen of dumbbell shapes with a length of 75 mm, width of 4 mm, and measured thickness of 0.20–0.30 mm were cut using a pneumatic press die cutter. All reported tensile measurements were triplicated.

## 4. Conclusions

In this study, we demonstrated the feasibility of preparing stable and uniform palm-based CNF-ZnO nanocomposites via a facile ultrasound route. The formation of nanocrystalline ZnO particles on the CNF matrix was confirmed by FTIR, XRD, FESEM, and TEM analysis. The ZnO particles were flake-like structures, and they were uniformly embedded onto the palm nanofibers with particles sizes ranging from 21 to 34 nm. The crystalline size of ZnO and its dispersion were found to be strongly governed by the pH conditions of the reaction solution. 

The in situ ultrasound route approach with pH variation confirmed the effectiveness of the size-controlled synthesis of ZnO on the cellulosic template. The ultrasonically-induced CNF-ZnO nanocomposite showed improved thermal stability as compared to pure CNF. Additionally, the resultant nanocomposite exhibited antibacterial efficacy against Gram-positive (MRSA) and Gram-negative (*S. typhi*) bacteria. The antibacterial properties suggested that the approach used in this study has the potential to solve the bottleneck issues of producing and applying palm- or waste-biomass-derived CNF in the biomedical sector. 

The incorporation of CNF-ZnO in the NR polymer network showed a significant improvement in the tensile strength and increased thermal stability compared with the neat NR and NR/CNF composite films. This work could pave the way for the use of less time-consuming synthesis techniques to fabricate CNF-ZnO hybrids without affecting the beneficial properties. Future research can be conducted on NR/CNF-ZnO composite films to further explore the antibacterial potential.

## Figures and Tables

**Table 1:** Formulation of NR composite films.

Ingredients	Neat NR	NR/CNF	NR/CNF-ZnO
35% NR (g)	100	100	100
60% Sulphur (g)	0.7	0.7	0.7
52% ZDEC (g)	0.6	0.6	0.6
61% ZnO (g)	0.7	0.7	0.7
Pure CNF (g)	-	5.0	-
CNF-ZnO (g)	-	-	5.0

## Data Availability

Not applicable.

## Supplementary Materials

The following are available online at https://www.mdpi.com/article/10.3390/ijms22115781/s1.
