# Supplementary material for: Facile Synthesis and Characterization of Palm CNF-ZnO Nanocomposites with Antibacterial and Reinforcing Properties
Source: Int J Mol Sci. 2021 May 28;22(11):5781. doi: 10.3390/ijms22115781 (PMC8197859; doi:10.3390/ijms22115781)
Supplement: Supplementary file 1 [file ijms-22-05781-s001.zip › ijms-1209155-supplementary.pdf]

Facile Synthesis and Characterization of Palm CNF-ZnO Nanocomposites with Antibacterial and Reinforcing Properties

Supplementary Information

Janarthanan Supramaniam [1], Darren Yi Sern Low [1], See Kiat Wong [1], Loh Teng Hern Tan [2,3], Bey Fen Leo [4, 5], Bey Hing Goh [6, 7, 8], Dazylah Darji [9], Fatimah Rubaizah Mohd Rasdi [9], Kok Gan Chan [10, 11]*, Learn Han Lee [3]* and Siah Ying Tang [1, 12, 13]*

[1] Chemical Engineering Discipline, School of Engineering, Monash University Malaysia, Bandar Sunway 47500, Selangor Darul Ehsan, Malaysia; janarthanan.supramaniam@monash.edu (J.S.); darrenl333.dl@gmail.com (D.Y.S.L.); see.wong@monash.edu (S.K.W.); patrick.tang@monash.edu (S.Y.T.)
[2] Clinical School Johor Bahru, Jeffrey Cheah School of Medicine and Health Sciences, Monash University Malaysia, Johor Bahru 80100, Johor Darul Ta'zim, Malaysia; loh.teng.hern@monash.edu (L.T.H.T.)
[3] Novel Bacteria and Drug Discovery Research Group (NBDD), Microbiome and Bioresource Research Strength (MBRS), Jeffrey Cheah School of Medicine and Health Sciences, Monash University Malaysia, Bandar Sunway 47500, Selangor Darul Ehsan, Malaysia; lee.learn.han@monash.edu (L.H.L.)
[4] Faculty of Medicine, University of Malaya, Kuala Lumpur 50603, Malaysia; beyfenleo@um.edu.my (B.F.L.)
[5] Nanotechnology and Catalysis Research Centre, University of Malaya, Kuala Lumpur 50603, Malaysia
[6] Biofunctional Molecule Exploratory Research Group (BMEX), School of Pharmacy, Monash University Malaysia, Bandar Sunway 47500, Selangor Darul Ehsan, Malaysia; goh.bey.hing@monash.edu (B.H.G.)
[7] College of Pharmaceutical Sciences, Zhejiang University, Hangzhou 310058, China
[8] Health and Well-Being Cluster, Global Asia in the 21st Century (GA21) Platform, Monash University Malaysia, Bandar Sunway 47500, Selangor Darul Ehsan, Malaysia
[9] Malaysian Rubber Board Engineering and Technology Division, RRIM, 47000 Sungai Buloh, Selangor Darul Ehsan, Malaysia; dazylah@lgm.gov.my (D.D.); rubaizah@lgm.gov.my (F.R.M.R.)
[10] Institute of Biological Sciences, Faculty of Science, University of Malaya, 50603, Kuala Lumpur, Malaysia; kokgan@um.edu.my (K.G.C.)
[11] International Genome Centre, Jiangsu University, Zhenjiang 212013, China
[12] Advanced Engineering Platform, School of Engineering, Monash University Malaysia, Bandar Sunway 47500, Selangor Darul Ehsan, Malaysia
[13] Tropical Medicine and Biology Platform, School of Science, Monash University Malaysia, Bandar Sunway 47500, Selangor Darul Ehsan, Malaysia
* Correspondence: kokgan@um.edu.my (K.G.C.); lee.learn.han@monash.edu (L.H.L.); patrick.tang@monash.edu (S.Y.T.); Tel.: +60-3-7967-7748 (K.G.C.); +60-3-5514-5887 (L.H.L.); +60-3-5514-4435 (S.Y.T.)

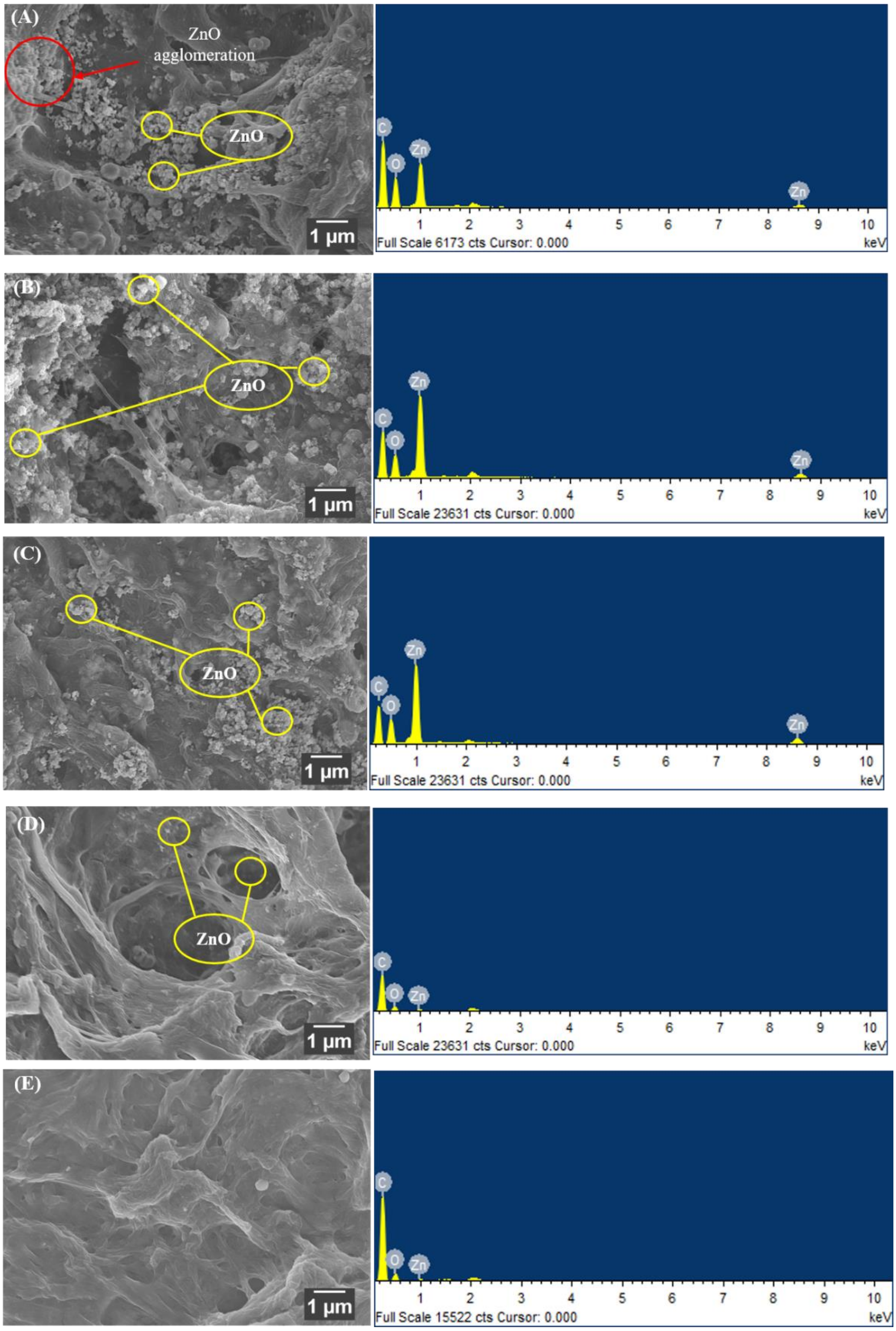

Figure S1: Morphology and EDX analysis of CNF-ZnO nanocomposite – (A) Control - pH 12.5, (B) pH 10, (C) pH 8, (D) pH 6, (E) pH 4.

The amount of zinc ions available used in this section was estimated using the equation below

$$Zn(C_2H_3O_2)_2.2H_2O \rightarrow Zn^{2+} + 2(C_2H_3O_2)^{-} + 2H_2O$$

The total zinc acetate dihydrate used in this experiment was 0.439 g dissolved in 200 ml of ultrapure water

The molecular weight of zinc acetate dihydrate was determined to be 219.498 g/mol

The molecular weight of elemental zinc = 65.37 g/mol

$$\frac{0.439\ \text{g}}{219.48\ \text{g/mol}} = \frac{0.439}{219.48}\ \text{mol} \rightarrow 0.002\ \text{mol}$$

$$\frac{0.439\ \text{g}}{200\ \text{ml x } 219.48\ \text{g/mol}} \times 1000\ \text{ml/l} = 0.01\ \text{mol/l}$$

$$\frac{0.01\ \text{mol/l} \times 65.37\text{g/mol}}{1.00\ \text{l}} = 0.6537\ \text{g/l} \rightarrow 653.7\ \text{mg/l}$$

Therefore, the estimated concentration of zinc ions used in this experiment was 653.7 mg/L. Using the ICP-OES method, the amount of zinc ion concentration on the CNF-ZnO nanocomposite was determined. Briefly, the ZnO-NP content in CNF before and after treatment was measured using ICP-OES (Perkin Elmer Optima 8000) with a 0.001 ppm metal ion detection limit. Initially the collected solution (filtrate) from the filter process of CNF-ZnO nanocomposite formation was used in this analysis. 1.00 ml of filtrate was diluted in 1000 ml of water and agitated via magnetic stirring at 500 rpm prior to the analysis. Approximately 10 ml of the diluted filtrate was transferred to a 50 ml plastic centrifuge tube and placed on a sample tray for the ICP-OES analysis. The Zn^{2+} content (Z) in CNF was determined from the following equation (1):

$$Z = C_{initial} - (C_{final} \times 1000) \qquad \textbf{(1)}$$

where, $C_{initial}$ is the concentration of initial zinc used to form the nanocomposite and C_{final} is the filtrate concentration. After the ICP-OES analysis, the amount of Zn^{2+} ions identified in the effluent was 0.009 ppm. Based on equation (1), the estimated amount of zinc presences on the fabricated nanocomposite was calculated to be 644.7 mg/l.

Sample Calculation

$$Z = 653.7\ \text{mg/l} - \left(0.009\ \text{mg/l} \times 1000\right) = 644.7\ \text{mg/l} \rightarrow 644.7\ \text{ppm}$$

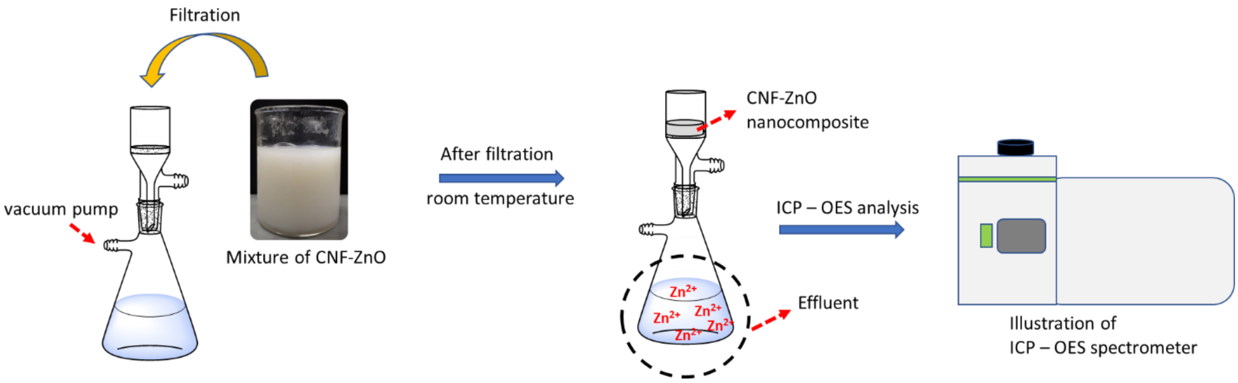

Figure S2: Method used to estimate the amount of Zn^{2+} presence in the synthesized CNF-ZnO nanocomposite.
